# Temporary hypotension and ventilation difficulty during endoscopic injection sclerotherapy for esophageal varices in a child with Fontan circulation: a case report

**DOI:** 10.1186/s40981-022-00538-z

**Published:** 2022-07-05

**Authors:** Nanako Yasutomi, Tatsuhiko Shimizu, Tomoyuki Kanazawa, Kazuyoshi Shimizu, Tatsuo Iwasaki, Hiroshi Morimatsu

**Affiliations:** 1 Department of Anesthesiology, Japanese Red Cross Kobe Hospital, Kobe, Japan; 2 Department of Anesthesiology and Resuscitology, Okayama University Hospital, 2-5-1 Shikata-cho, Kita-ku, Okayama, 700-8558 Japan

**Keywords:** Congenital heart disease, Fontan circulation, Esophageal varices, Endoscopic injection sclerotherapy

## Abstract

**Background:**

Endoscopic procedures are rarely performed in children with congenital heart disease (CHD); therefore, the associated complications are unknown. We report an abrupt change in circulatory and respiratory condition during endoscopic injection sclerotherapy for esophageal varices.

**Case presentation:**

A 9-year-old boy with a history of total anomalous pulmonary venous connection (TAPVC) repair and Fontan procedure for asplenia and a single ventricle with TAPVC underwent endoscopic injection sclerotherapy under general anesthesia for esophageal varices. Systolic blood pressure decreased from 70 to 50 mmHg following a sclerosant injection; a second injection reduced his peripheral oxygen saturation from 93 to 79% secondary to ventilation difficulty. Although we suspected anaphylaxis intraoperatively, postoperative imaging suggested that balloon dilation performed to prevent sclerosing agent leakage caused compression of the pulmonary venous chamber and trachea owing to the anomalous intrathoracic organ anatomy.

**Conclusion:**

Thorough understanding of the complex anatomy is important before performing endoscopic procedures in children with CHD to preoperatively anticipate possible intraoperative complications and select the optimal therapeutic approach and anesthesia management.

## Background

In recent years, surgical outcomes for patients with congenital heart disease (CHD) have improved, and non-cardiac surgery for postoperative children with CHD have become popular. However, endoscopic therapy is rare for children with CHD, and it is unknown what complication would be happened during procedure. We report a rare case who had severe hypotension and respiratory failure during endoscopic injection sclerotherapy (EIS) for esophageal varices (EV) in a patient with CHD.

## Case presentation

A 9-year-old boy (height 118 cm; weight 18 kg) with asplenia, dextrocardia, single atrium, single ventricle, common atrioventricular valve, and total anomalous pulmonary venous connection (TAPVC) had undergone a TAPVC repair and a Fontan surgery with fenestration by 3-year-old, in addition, multiple coil embolizations for major aortopulmonary collateral arteries. He had developed protein losing enteropathy and plastic bronchitis after Fontan surgery. Furthermore, he experienced endoscopic esophageal variceal ligation (EVL) for EV twice at 7-year-old without any adverse events.

This time, he was rushed to the emergency room at another hospital for massive hematemesis. His condition was stabilized by conservative treatment such as a blood transfusion, and he was transferred to our hospital. An emergency gastroscopy revealed moniliform moderate varices with redness extending from the gastric cardia to the upper esophagus; however, there was no active bleeding. Subsequent cardiac angiography revealed the central venous pressure of 14 mmHg, and there was no indication for a catheterization procedure. Thus, EIS under general anesthesia was scheduled for the treatment of EV.

His preoperative blood pressure (BP) was 84/46 mmHg, heart rate (HR) was 91 beat per minute, and peripheral oxygen saturation (SpO_2_) was 96% at 1 L/min of oxygen through a nasal cannula. Preoperative electrocardiogram showed sinus rhythm, and chest X-ray indicated a cardiothoracic ratio of 44.5%. Transthoracic echocardiography revealed ejection fraction of 56% with mild common atrioventricular valve regurgitation, and blood tests showed mildly decreased hemoglobin (12.5 g/dL) and elevated hepatobiliary enzymes (total bilirubin 2.8 mg/dL, γ-glutamyl transpeptidase 160 IU/L).

After the induction of general anesthesia with midazolam 3 mg, fentanyl 50 μg, remifentanil 0.3 μg /kg/min, and rocuronium 20 mg, he was intubated, and we maintained general anesthesia with sevoflurane 1.5–2% and remifentanil 0.05–0.15 μg/kg/min. The ventilator mode was set to pressure-controlled ventilation, with driving pressure of 10–13 cmH_2_O, positive end-expiratory pressure of 5 cmH_2_O, respiratory rate of 20/min, and fraction of inspiratory oxygen (F_I_O_2_) 0.47. He was monitored via electrocardiogram, SpO_2_, continuous arterial BP, and capnometry.

We used an OLYMPUS GIF TYPE Q260J endoscope with a diameter of 9.9 mm and 5% ethanolamine oleate with iopamidol as the sclerosing agent (SA) for EIS. We attached an EV balloon (MD-690, Create medic, Kanagawa, Japan) near the tip of the endoscope and dilated the balloon with 20 ml of air just before injecting the SA to prevent it from leaking out. The SA was injected into the varicose vein with a 25G puncture needle under X-ray fluoroscopy.

**Fig. 1:**
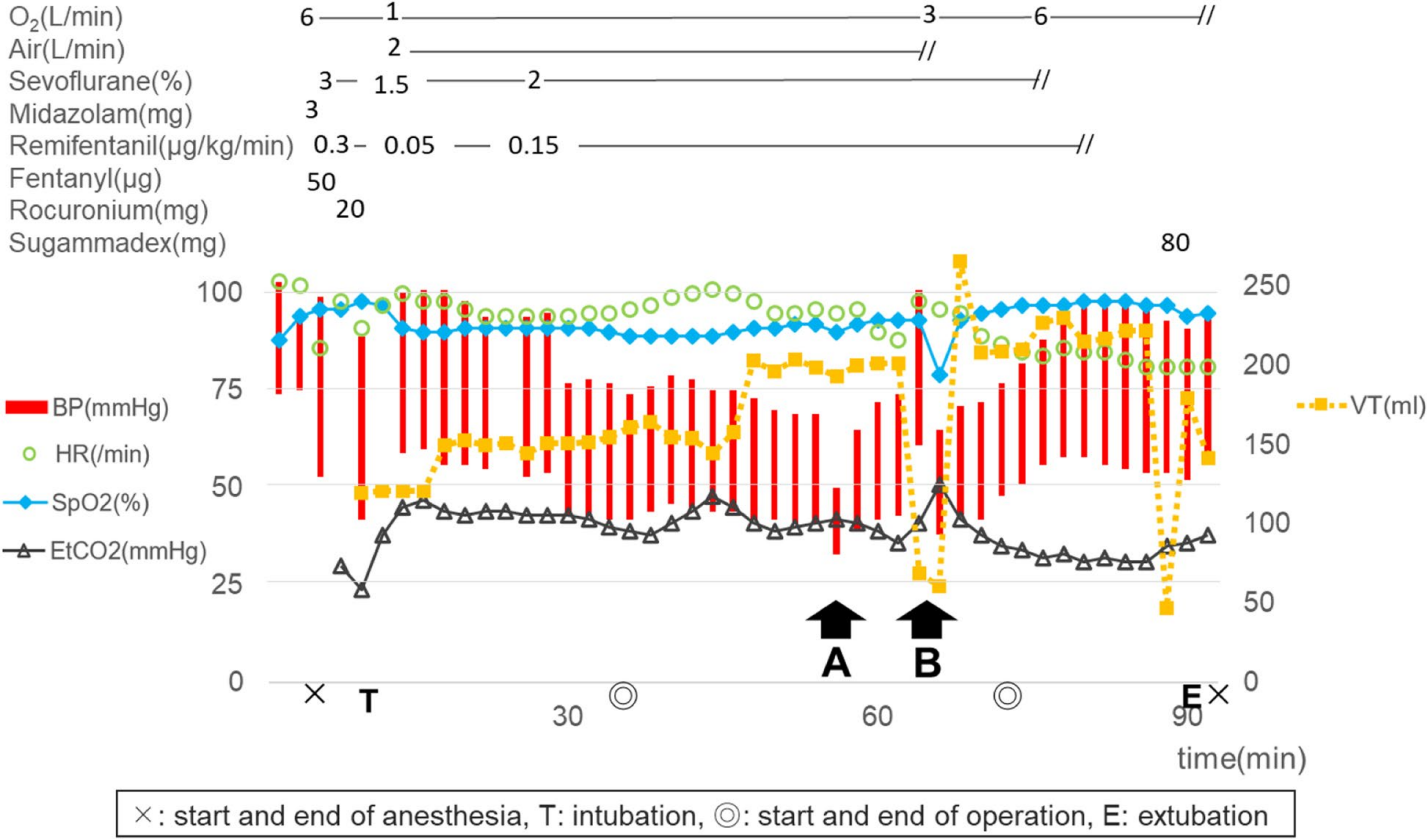
Anesthesia record. An abrupt decrease of blood pressure (**A**), tidal volume, and SpO_2_ (**B**) occurred corresponding to the timing of inflation of the balloon at the tip of the endoscope and injection of the sclerosing agents into the varicose veins in gastric cardia (**A**) and in the esophagus (**B**), respectively. BP: blood pressure, HR: heart rate, VT: tidal volume

After the procedure started, his vital signs were stable. However, at the time of the SA injection into the varicose vein in the gastric cardia a short time after the balloon was dilated, his systolic BP decreased from 70 to 50 mmHg (Fig. 1A). At first, we suspected anaphylaxis due to the SA partly because we did not know about the balloon dilation; however, there was no change in HR or tidal volume (VT) and also no allergic findings such as skin reddening. After that, BP gradually recovered when the balloon was deflated about 3 min later. Next, the SA was injected into the esophageal varices about 5 cm cephalad from the first injection point in the same way. Then, immediately after the balloon dilation, the VT decreased from 200 to 60 ml, SpO_2_ decreased from 93 to 79%, end-tidal CO_2_ (EtCO_2_) increased from 36 to 51 mmHg, and HR increased from 88 to 98/min (Fig. 1B). We started manual ventilation with 100% oxygen right away. That restored SpO_2_; however, ventilation was inadequate until VT and EtCO_2_ were recovered by the balloon deflation a few minutes later. Thereafter, his respiratory and circulatory dynamics were stable, and he was extubated in the operating room.

Although he had the good postoperative course, EVL was performed for rebleeding from compensatory enlarged gastric varices on the 11th postoperative day (POD), followed by a percutaneous fenestration re-creation on the 26th POD, and he was transferred to another hospital for rehabilitation on the 55th POD.

## Discussion

Regarding gastroesophageal varices after CHD surgery, Kiesewetter et al. reported that 7 of 12 patients in the distant stage after Fontan surgery had congestive cirrhosis, and 4 of them had gastroesophageal varices [1]. Whereas there are a few reports of EIS for EV in non-CHD patients [2–5], there are no reports in postoperative CHD patients, nor are there any reports of circulatory and/or respiratory complications during the procedure.

**Fig. 2:**
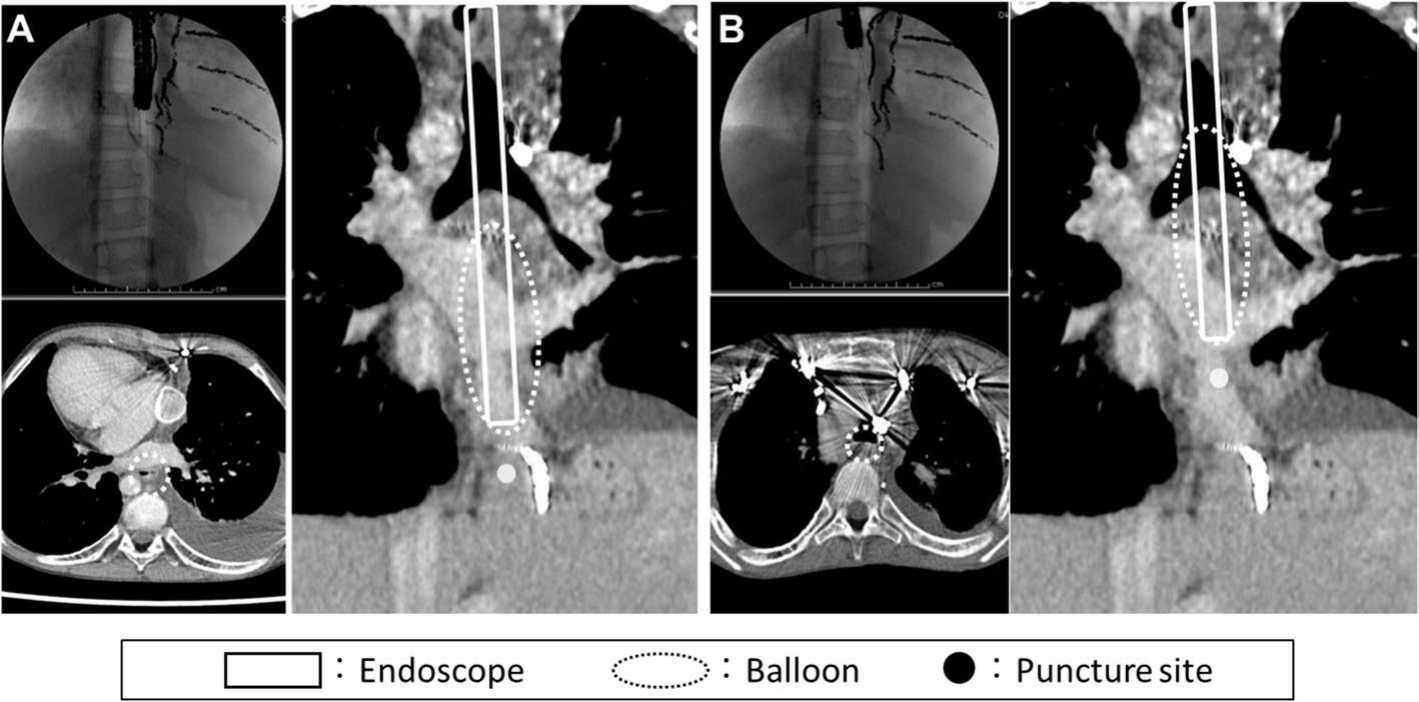
Intraesophageal balloon position inferred from perioperative CT and fluoroscopic images during injection of sclerotic agents into the varicose vein of the gastric cardia (**A**) and of the esophagus (**B**). Intraoperative fluoroscopic image (upper left), perioperative axial (lower left), and coronal (right) CT images. Note the balloon located behind the common pulmonary venous cavity (**A**) and the coils in front of the tracheal bifurcation (**B**)

**Fig. 3:**
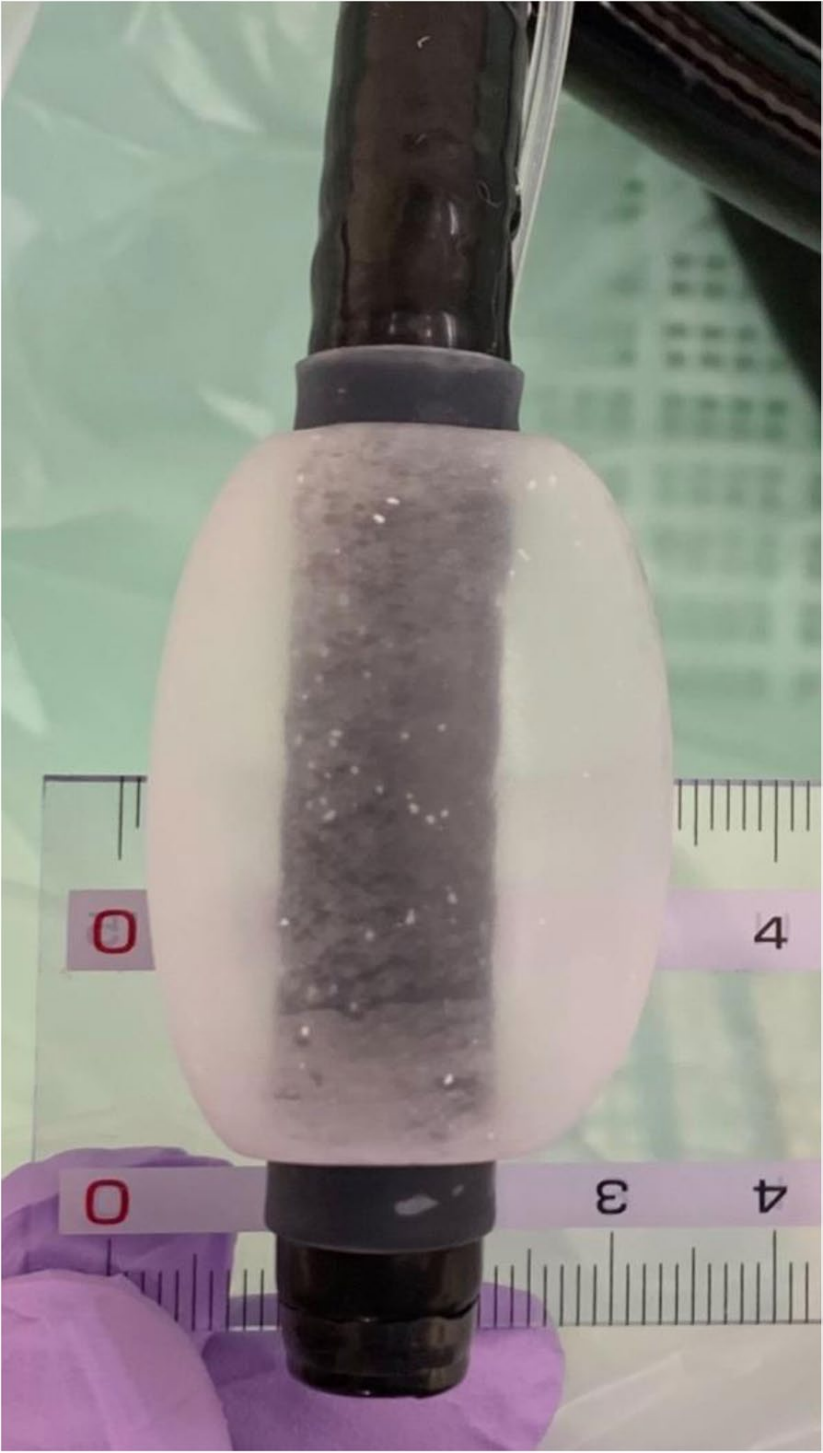
Postoperative verification of the balloon size. The maximum diameter of the balloon was 32 mm when 20 ml of air was injected into the esophageal varices balloon

We compared the preoperative CT images, intraoperative fluoroscopic images, and esophageal balloon size to find the cause of hypotension and ventilatory failure in this case. The position of the puncture needle and balloon during injection of the SA is supposed to be as shown in Fig. 2. The EV balloon attached to the tip of the endoscope in this case is described in the package insert as having a diameter of 32 mm when 23 ml of air is injected. During the procedure, the injected volume of air was reduced to 20 ml at the discretion of the surgeon in consideration of the patient’s physique; however, the maximum diameter was also 32 mm when 20 ml of air was injected in a postoperative verification using the same type of balloon (Fig. 3). Therefore, it is better to measure and understand the change in balloon diameter with the amount of air before procedure.

We assessed that cardiac output was dropped by balloon compressing of the common pulmonary venous cavity during the first injection, which resulted in decreased BP (Fig. 2A). In the second infusion, we considered that inadequate ventilation due to compression of the tracheal bifurcation by the balloon induced a decrease in SpO_2_ and an increase in EtCO_2_, and direct stimulation of the trachea caused an increase in BP and HR (Fig. 2B). We also confirmed with the surgeon that the timing of balloon dilation had been consistent with these events.

**Fig. 4:**
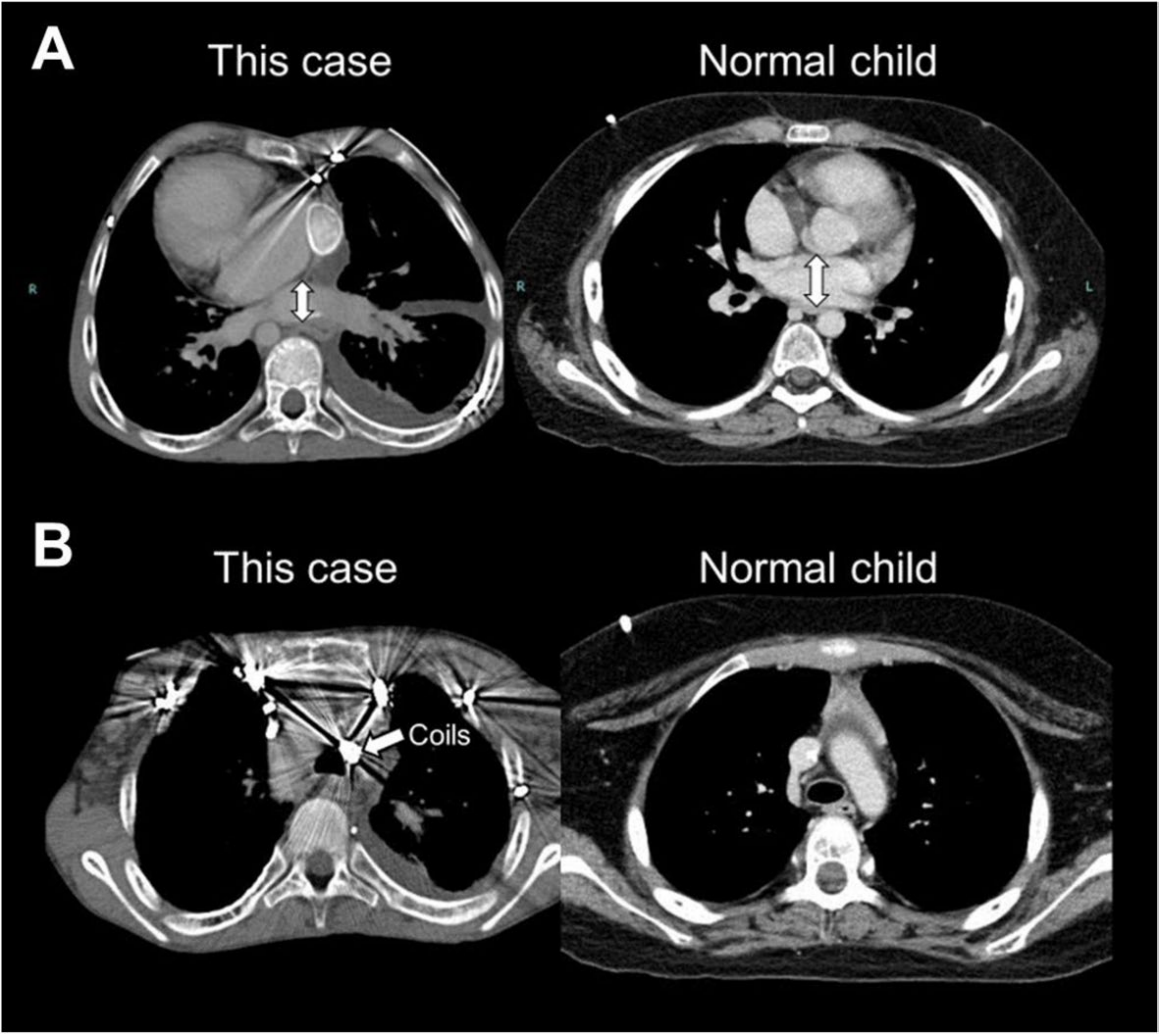
Transverse CT image at the levels of the common pulmonary venous cavity (**A**) and tracheal bifurcation (**B**) in this patient (left) and in a normal child (right). Note the smaller anterior-posterior diameter of the common pulmonary venous cavity in this case (left) compared with that of the left atrium in a normal child (right) (**A**) and the coils for embolizing the major aortopulmonary collateral arteries in front of the tracheal bifurcation (left) (**B**)

There have been no reports of these same phenomena occurring in children without CHD. We thought that the reason was the anatomical difference between this patient and normal children without anatomic deformities. In this patient, the common pulmonary venous cavity after TAPVC repair was shorter in anteroposterior diameter than the left atrium of normal children (Fig. 4A). Therefore, a decrease in preload due to balloon compression and a concomitant BP decrease was likely to occur. On the other hand, the coils embolized for major aortopulmonary collateral arteries were located in front of the tracheal bifurcation in this patient (Fig. 4B). We assessed that was why the trachea might be more easily compressed in spite of trachea cartilages by being sandwiched between the esophageal balloon and the coils. Besides, this patient had experienced EVL twice at 7-year-old without adverse events. The reason was presumed that EVL is an endoscopic procedure in which a rubber band is placed over the varicose vein to stop the bleeding without a balloon, as opposed to EIS.

In order to properly respond to such complications of respiratory and circulatory changes, the following points should be mentioned. First, it is important to understand the complex anatomy preoperatively and anticipate possible complications. In addition, it is necessary to monitor an invasive arterial BP, and good communication should be maintained with the surgeon to understand the timing and duration of balloon inflation, since lack of information about balloon manipulation during this procedure was one of the factors that prevented us from identifying the causes. They would permit to take countermeasures against hypotension as in pattern of Fig. 1A, such as preloading with intravenous fluids and administering vasoconstrictors, and against ventilatory failure as in pattern of Fig. 1B, such as increasing F_I_O_2_ in advance. If we increase the inspiratory force against a decrease in VT due to airway obstruction, the high intrathoracic pressure by check valve would induce a BP decrease, especially in Fontan circulation. Therefore, if airway obstruction is predicted, it may be better to only prevent a significant SpO_2_ drop by raising F_I_O_2_ in advance and tolerate temporary hypoventilation for a while, which may reduce the risk of secondary complications. However, in cases with pulmonary hypertension, it may be critical due to increased pulmonary vascular resistance by hypoventilation-induced hypercapnia, so we think that it is necessary to discuss the treatment strategy with the surgeon.

In conclusion, understanding each anatomy in CHD and anticipating possible intraoperative complications preoperatively may allow for appropriate treatment selection and anesthetic management.

## Data Availability

Not applicable

## Abbreviations

CHD: Congenital heart disease
TAPVC: Total anomalous pulmonary venous connection
EIS: Endoscopic injection sclerotherapy
EV: Esophageal varices
EVL: Endoscopic variceal ligation
BP: Blood pressure
HR: Heart rate
SpO_2_: Peripheral oxygen saturation
F_I_O_2_: Fraction of inspiratory oxygen
SA: Sclerosing agent
VT: Tidal volume
EtCO_2_: End-tidal CO_2_
POD: Postoperative day
